# Differential antibody response to the *Anopheles gambiae* gSG6 and cE5 salivary proteins in individuals naturally exposed to bites of malaria vectors

**DOI:** 10.1186/s13071-014-0549-8

**Published:** 2014-11-28

**Authors:** Cinzia Rizzo, Fabrizio Lombardo, Raffaele Ronca, Valentina Mangano, Sodiomon Bienvenu Sirima, Issa Nèbiè, Gabriella Fiorentino, David Modiano, Bruno Arcà

**Affiliations:** Department of Public Health and Infectious Diseases, Division of Parasitology, Sapienza University, Piazzale Aldo Moro 5, 00185 Rome, Italy; Department of Biology, “Federico II” University, Naples, Italy; Centre National de Recherche et de Formation sur le Paludisme, Ouagadougou, Burkina Faso

**Keywords:** *Anopheles gambiae*, Salivary proteins, Immune response, IgG, IgG1, IgG4, Marker of exposure, *Plasmodium* transmission, Malaria epidemiology

## Abstract

**Background:**

Mosquito saliva plays crucial roles in blood feeding but also evokes in hosts an anti-saliva antibody response. The IgG response to the *Anopheles gambiae* salivary protein gSG6 was previously shown to be a reliable indicator of human exposure to Afrotropical malaria vectors. We analyzed here the humoral response to the salivary anti-thrombin cE5 in a group of individuals from a malaria hyperendemic area of Burkina Faso.

**Methods:**

ELISA was used to measure the anti-cE5 IgG, IgG1 and IgG4 antibody levels in plasma samples collected in the village of Barkoumbilen (Burkina Faso) among individuals of the Rimaibé ethnic group. Anti-gSG6 IgG levels were also determined for comparison. *Anopheles* vector density in the study area was evaluated by indoor pyrethrum spray catches.

**Results:**

The cE5 protein was highly immunogenic and triggered in exposed individuals a relatively long-lasting antibody response, as shown by its unchanged persistence after a few months of absent or very low exposure (dry season). In addition cE5 did not induce immune tolerance, as previously suggested for the gSG6 antigen. Finally, IgG subclass analysis suggested that exposed individuals may mount a Th1-type immune response against the cE5 protein.

**Conclusions:**

The anti-cE5 IgG response is shown here to be a sensitive indicator of human exposure to anopheline vectors and to represent an additional tool for malaria epidemiological studies. It may be especially useful in conditions of low vector density, to monitor transiently exposed individuals (i.e. travellers/workers/soldiers spending a few months in tropical Africa) and to evaluate the impact of insecticide treated nets on vector control. Moreover, the gSG6 and cE5 salivary proteins were shown to trigger in exposed individuals a strikingly different immune response with (i) gSG6 evoking a short-lived IgG response, characterized by high IgG4 levels and most likely induction of immune tolerance, and (ii) cE5 eliciting a longer-living IgG response, dominated by anti-cE5 IgG1 antibodies and not inducing tolerance mechanisms. We believe that these two antigens may represent useful reagents to further investigate the so far overlooked role of *Anopheles* saliva and salivary proteins in host early immune response to *Plasmodium* parasites.

**Electronic supplementary material:**

The online version of this article (doi:10.1186/s13071-014-0549-8) contains supplementary material, which is available to authorized users.

## Background

The saliva of hematophagous arthropods is a complex cocktail of bioactive molecules whose main function is to facilitate blood acquisition by targeting host hemostatic, inflammatory and immune responses [1,2]. Although vector saliva originally evolved to assist blood feeding, its injection into the vertebrate skin modulates host immune responses, which in turn may affect transmission or establishment of pathogens [3-5]. In addition individuals repeatedly bitten by arthropods carry circulating anti-saliva antibodies that can be exploited as a tool to evaluate human exposure to disease vectors as diverse as ticks, sand flies, triatomines, tsetse flies and mosquitoes [6,7]. According to their highly adaptive value, and under the selective pressure of the host immune system, salivary proteins of blood-feeding arthropods evolve at a very fast rate as clearly shown in sand flies and mosquitoes [8,9]. Perhaps also as a consequence of this rapid divergence transcriptome analyses revealed that mosquito saliva includes not only a relatively large number of family-specific proteins, *i.e*. found in culicids but in no other blood-feeding arthropods, but also genus-specific salivary proteins, *i.e*. uniquely found in the saliva of either anopheline or culicine mosquitoes [10].

We have previously studied the human antibody response to the anopheline-specific gSG6 salivary protein in a cohort from a malaria hyperendemic area of Burkina Faso, i.e. in individuals naturally exposed to bites of Anopheles mosquitoes (mainly *Anopheles gambiae* and *Anopheles funestus*). In *An. gambiae* gSG6 is specifically found in the saliva of adult female mosquitoes [11] and the protein must play some crucial role in blood feeding since its depletion by RNAi prolongs probing time and affects blood feeding efficiency [12]. Analysis of the IgG antibody response to the gSG6 recombinant protein indicated it is a suitable serological marker of human exposure to African malaria vectors in different epidemiological settings [13-16] and similar results have been obtained with the less sensitive gSG6-P1 peptide [17-19]. The availability of simple immunoassays to measure human-vector contact represents a very useful tool for the evaluation of malaria transmission intensity and disease risk, especially in settings where the use of classical entomological methods is difficult or unfeasible (low malaria transmission, low/reduced vector density, logistics, etc.). Moreover, since serology with parasite antigens is commonly used in malaria studies [20] the parallel use of salivary antigens to obtain information on exposure to vectors appears very convenient. In this respect the availability of additional salivary antigens enriching the serological toolbox would be very valuable, allowing us to overcome potential problems linked to individual variation of the immune response and providing reagents with different immunogenicity, which could be very useful to detect variation in vector exposure in different epidemiological settings.

In addition to gSG6 we have also expressed and purified in recombinant form another *An. gambiae* salivary protein that is only found in mosquitoes of the Anophelinae subfamily, *i.e*. it is not found in the saliva of culicine mosquitoes or other blood-feeding arthropods and shows no similarity to any other known polypeptide. This protein was originally named cE5 [21] and then found to be a member of the anophelin family of anti-thrombin peptides [22,23]. Initial indications showed that the cE5 protein was immunogenic in humans, encouraging further investigation of the humoral immune response to this protein in exposed individuals. We report here an analysis of the IgG, IgG1 and IgG4 antibody responses to the *An. gambiae* salivary proteins cE5 in a group of individuals from a malaria hyperendemic area of Burkina Faso. Comparison of the humoral response to the cE5 and gSG6 salivary proteins clearly indicates that these two proteins evoke substantially different responses in individuals exposed to bites of anopheline mosquitoes.

## Methods

### Study area and subjects

The study was conducted in the village of Barkoumbilen, located in a rural malaria hyperendemic area of Burkina Faso (≈35 kilometers NE of Ouagadougou) and inhabited by the two sympatric ethnic groups Mossi and Rimaibé. These two groups are both Sudanese Negroid populations with a remarkably similar response to parasite antigens and susceptibility to malaria as opposed to individuals of the sympatric ethnic group Fulani, who are less susceptible to malaria and exhibit higher humoral response to both parasite and mosquito salivary antigens [15,24-26]. *Plasmodium falciparum* transmission is intense in the area, especially during the June–October rainy season (Entomological Inoculation Rate (EIR) >100/person/year) and malaria prevalence is very high, with most of malaria infections (≈95%) caused by *P. falciparum*. Infection rates in both the Mossi and Rimaibé groups were of 60%-90% (depending on age) during the high-transmission rainy season and of 40-80% in the low-transmission dry season. Samples were collected during three cross-sectional surveys carried out at the beginning (August 1994) and the end (October 1994) of the high-transmission season, as well as during the following low-transmission season (March 1995). *Plasmodium falciparum* inoculation rates were based on indoor vector collections and immunoenzymatic estimation of circumsporozoite protein positivity as previously reported [27].

### Ethics statement

The study protocol was approved by the Technical Committee of the Centre National de Lutte contre le Paludisme of the Ministry of Health of Burkina Faso. Oral informed consent for multiple immuno-parasitological, clinical and entomological surveys was obtained from the Rimaibé community living in Barkoumbilen. The samples utilized for this analysis have been collected in the period from August 1994 to March 1995 in the frame of a larger epidemiological study performed in Burkina Faso [25]. At that time a national ethic committee was not yet in place and written consent was not requested. Therefore, the study protocol and the oral informed consent were approved by the Institutional Review Board, i.e. by the Technical Committee of the Centre National de Lutte contre le Paludisme of the Ministry of Health of Burkina Faso. Individual oral consent was obtained from all adults and from children’s parents or legal representatives. In June-July 1994, prior to the start of the surveys, meetings were held in the villages of the study area to explain, in the local languages, the objectives of the study, the procedures involved and to answer questions from the residents.

### Entomological data

Entomological measures were based on indoor pyrethrum spray catches carried out monthly between August and November 1994 and in March 1995 (12 catches/month). In the study area the main malaria vectors were *An. gambiae*, *An. arabiensis* and *An. funestus*, with the members of the *An. gambiae* species complex (i.e. *An. gambiae* and *An. arabiensis*) representing, on average, approximately 90% of the indoor-resting anopheline mosquitoes. As previously reported, the number of anopheles/person/night ±95% CI in the village of Barkoumbilen were 6.3 ± 1.5 (August 1994), 12.1 ± 5.4 (September-October 1994) and 0.4 ± 0.2 (March 1995) [15]; additional details on the study site and parasitological aspects can be found elsewhere [24,25,27].

### Plasma samples

Blood samples were collected in K3-EDTA sterile tubes. Within 3–4 hr after bleeding, the plasma was transferred and kept at −20°C until serological tests were done. All samples analyzed in this study were collected in the village of Barkoumbilen among individuals of the Rimaibé ethnic group. A total of 207 human plasma samples collected during the Aug 1994 survey (1–74 years old, average age ±95% CI = 22.7 ± 16.9) were used for (i) the comparative determination of the anti-cE5 and anti-gSG6 IgG levels and for (ii) the measurement of anti-cE5 IgG1 and IgG4 antibodies. The seasonal variation of the anti-cE5 IgG response was measured in samples collected during three different surveys: Aug 1994 (n = 117, 1–70 years old, 17.2 ± 3.0), Oct 1994 (n = 121, 1–70 years old, 16.8 ± 3.1) and Mar 1995 (n = 121, 1–70 years old, 16.0 ± 2.9). Fifty-nine plasma samples from randomly selected individuals (1–69 years old, 26.3 ± 16.1) who referred to a public hospital of Rome’s municipality for routine blood tests were used as unexposed controls.

### Enzyme-linked immunosorbent assays

ELISA was performed according to standard procedures. The gSG6 and cE5 proteins were expressed and purified as previously described [15,22]. Flat-bottom, 96-well plates (Nunc Maxisorp, M9410) were coated overnight with 50 μl of either gSG6 or cE5 at 5 μg/ml diluted in coating buffer (15 mM Na_2_CO_3_, 35 mM NaHCO_3_, 3 mM NaN_3_, pH 9.6). Wells were washed four times with PBST (0.05% Tween-20 in 1x PBS), blocked for 3 hours at 25°C (150 μl 1% w/v skimmed dry milk in PBST), washed again as above and incubated overnight at 4°C with 50 μl of plasma diluted 1:100 (IgG) or 1:20 (IgG1 and IgG4). Plates were then washed as above and incubated for 3 hours at 25°C with 100 μl of polyclonal rabbit anti-human IgG/HRP (Dako P0214, dilution 1:5000 in blocking buffer) or 100 μl of sheep anti-human IgG1/HRP (Binding Site AP006, dil. 1:1000) or 100 μl of sheep anti-human IgG4/HRP (Binding Site AP009, dil. 1:1000). After washing the colorimetric reaction was carried out with 100 μl of o-phenylenediamine dihydrochloride (OPD, Sigma P8287; 15 min, 25°C in the dark). Reactions were terminated by adding 25 μl of 2 M H_2_SO_4_. OD_492_ were determined using a microplate reader (Biotek Synergy HT). IgG1 and IgG4 OD levels were converted to concentrations (ng/ml) using standard curves set up as follows. As capturing factors goat anti-human IgG (5 μg/ml; Jackson ImmunoResearch Laboratories Inc, PA, USA) or mouse anti-human IgG4 (2 μg/ml; BD Pharmingen, USA) were used for coating (50 μl coating buffer, overnight at 4°C). After washing, blocking and washing again as above wells were incubated overnight at 4°C with serial dilutions, from 1 μg/ml to 0.0078 μg/ml (1 → 0.5 → 0.25 → 0.125 → 0.0625 → 0.03125 → 0.0156 → 0.0078), of purified native human IgG1 or IgG4 (ABD Serotec, Kidlington, Oxford, UK) in 50 μl of blocking reagent. Incubation with anti-human IgG1/HRP or IgG4/HRP and colorimetric detection were performed as described above.

### Data analysis

IgG, IgG1 and IgG4 levels were determined by analyzing samples in duplicate with the antigen and once without antigen (coating buffer only). Final OD was calculated as the mean OD value with antigen minus the OD value without antigen. Serial dilutions of a pool of plasma samples were added to each plate as standard curve to normalize experimental variability among plates. The two-fold serial dilutions were 1:30 to 1:960 for IgG determination and 1:4 to 1:512 for IgG1 and IgG4. Intra and inter assay variation of standard samples was always below 20%. The very few samples whose duplicates showed a coefficient of variation >20% were not included in the analysis. Multiple comparisons were performed by the Kruskal-Wallis test. The Mann–Whitney U test was used to compare two independent groups and the Wilcoxon matched-pairs test was used for comparison of two paired groups. Proportions were compared by the Fisher’s exact test. Receiver Operating Characteristic (ROC) curve analysis were used to obtain information on the accuracy of the assays and to calculate cut-off values for seropositivity as follows: Aug 1994 IgG cE5 > 0.0705 (95.19% sensitivity, 96.61% specificity, area under curve 0.9808), Aug 1994 IgG gSG6 > 0.0645 (77.88% sensitivity, 84.75% specificity, area under curve 0.8610), Aug 1994-Oct 1994-Mar 1995 IgG cE5 > 0.06786 (92.48% sensitivity, 94.12% specificity, area under curve 0.9684). All statistical analyses were performed using GraphPad Prism 6.0 statistical software (GraphPad Software Inc., La Jolla, CA).

## Results

### cE5 immunogenicity

**Figure 1:**
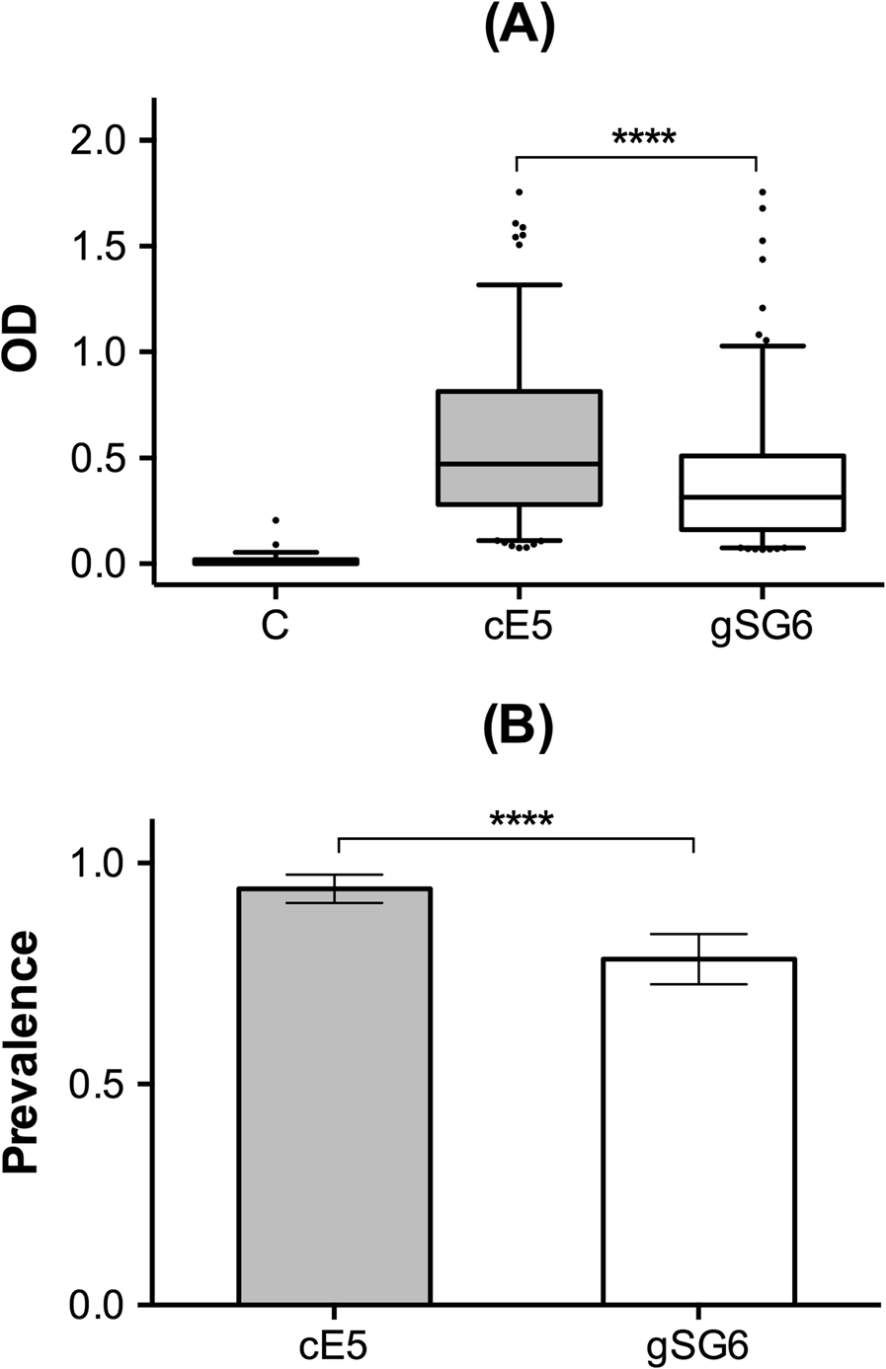
**IgG antibody response to the** ***An. gambiae*** **cE5 and gSG6 proteins.** Comparison of the anti-cE5 and anti-gSG6 IgG responses among exposed individuals during the malaria transmission season (survey Aug 1994, n = 207, average age ±95% CI = 22.7 ± 16.9). **(A)** Box plots of OD values among responders to both cE5 and gSG6 antigens (n = 156). Boxes display median values, 25th and 75th percentiles. Whiskers represent 5–95 percentiles and dots the outliers. The IgG response to the cE5 protein in non-exposed individuals (n = 59, average age ±95% CI = 26.3 ± 16.1) is shown as control (C). IgG levels are expressed as OD_492_. P value determined by the Wilcoxon matched-pairs signed rank test (****, p < 0.0001). **(B)** Seroprevalence of anti-cE5 and anti-gSG6 IgG antibodies. Error bars indicate 95% confidence intervals. P value determined by Fisher’s exact test (****, p < 0.0001).

Following initial indications of cE5 immunogenicity to humans we measured IgG antibody levels in samples collected during the malaria transmission season (Aug 1994, n = 207) in the village of Barkoumbilen, Burkina Faso. As a comparison we also analyzed the response of the same individuals to the *An. gambiae* salivary protein gSG6, whose antigenic properties have been previously investigated [14-16,26]. IgG antibodies against the cE5 protein were elevated in exposed individuals but very low, or absent, in unexposed control subjects (Figure 1A). In addition, anti-cE5 IgG levels were significantly higher than anti-gSG6 IgG levels, as indicated by a comparison of the OD values among responders. Similarly, the seroprevalence was significantly higher for the cE5 (94%) than for the gSG6 antigen (78%, Figure 1B). According to these observations the *An. gambiae* salivary protein cE5 appeared to be immunogenic in humans and to evoke in exposed individuals an IgG response of higher intensity in comparison to gSG6.

### IgG response to the cE5 and gSG6 proteins in different age groups

**Figure 2:**
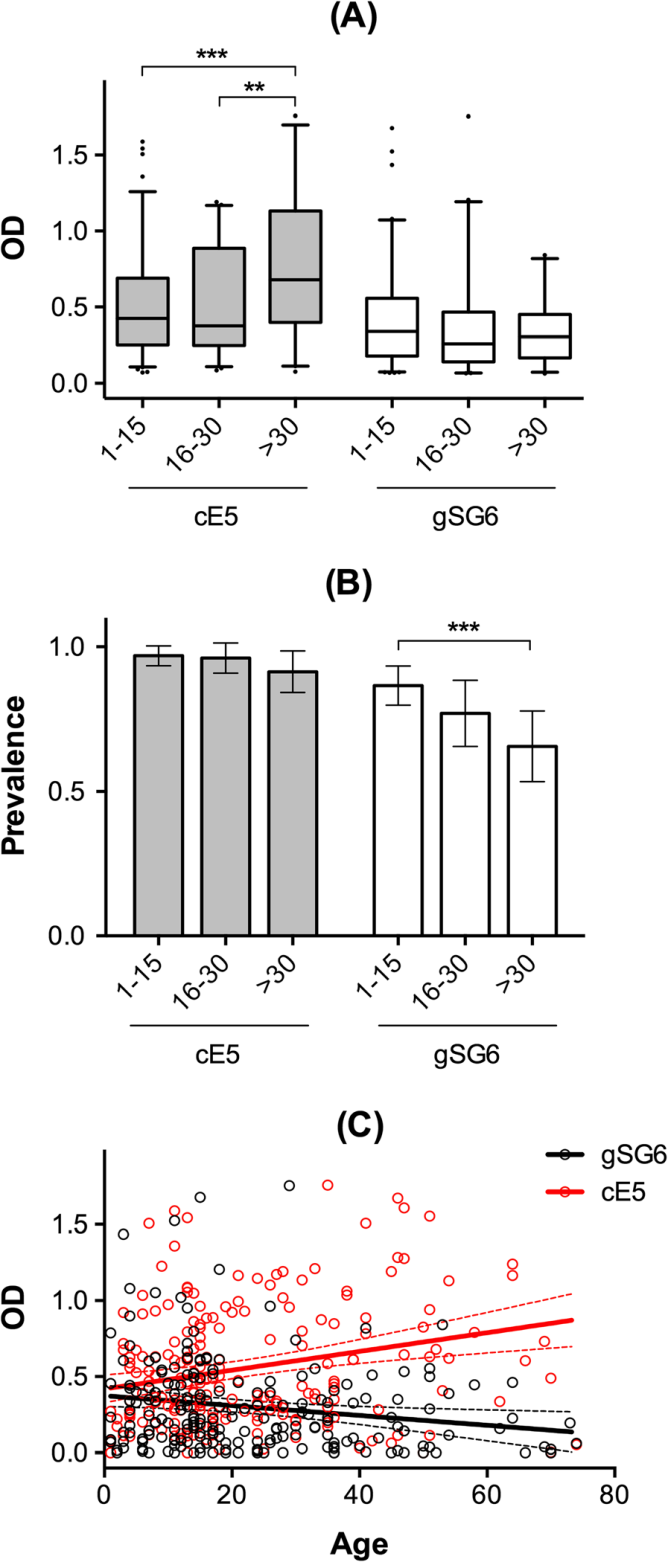
**Comparison of the IgG response to cE5 and gSG6 in different age groups. (A)** Box plots of OD values among responders to cE5 (1–15 n = 94; 16–30 n = 50; >30 n = 53) and to gSG6 (1–15 n = 84; 16–30 n = 40; >30 n = 38) in the three age groups as indicated. Boxes, whiskers and dots as in Figure 1. IgG levels are expressed as OD_492_. P values determined by the Kruskal-Wallis (cE5 P = 0.001; gSG6 ns) and the Mann–Whitney tests (***, p < 0.001; **, p < 0.01). **(B)** Seroprevalence of anti-cE5 and anti-gSG6 IgG antibodies in the different age groups (1–15 n = 97; 16–30 n = 52; >30 n = 58). Error bars and P value as in Figure 1 (***, p < 0.001). **(C)** Scatter plot reporting the IgG antibody response to cE5 (red) and to gSG6 (black) as function of age (n = 207). The best-fit lines (solid) and 95% CI (broken lines) are shown. Note that one data point is outside the axis limits.

The IgG antibody response to the salivary protein gSG6 was previously found to be higher in young children than in adults or elderly people. As a possible explanation of this age-dependent pattern it was suggested that the continued and intense exposure to bites of anopheline mosquitoes induced immune tolerance to this antigen [15,26]. Fully consistent results where obtained in the present study where median anti-gSG6 IgG levels and seroprevalence were higher in 1–15 years old individuals than in the other two age groups (Figure 2A-B). On the contrary, a different trend was observed for the cE5 salivary protein: anti-cE5 IgG levels were significantly higher in individuals older than 30 years although no difference was observed when seroprevalence was considered, most likely because of the very high values found in all age groups (>90%, Figure 2A-B). A consistent pattern was also observed when the youngest age group was further stratified in very young (1–5 years old), young (6–10 years old) and older (11–15 years old) children (Additional file 1: Figure S1). The difference in the IgG response to the two antigens studied appeared especially striking when individual OD values were reported as a function of age (Figure 2C): the two divergent best fit lines (significantly non-zero: gSG6, p = 0.0111; cE5, p = 0.0003) clearly show opposite trends for the IgG response to gSG6 and cE5. This age-related pattern of the anti-cE5 IgG response suggests that the *An. gambiae* cE5 does not induce immune tolerance, even after prolonged and intense exposure.

### Anti-cE5 IgG1 and IgG4 subclasses in exposed individuals

**Figure 3:**
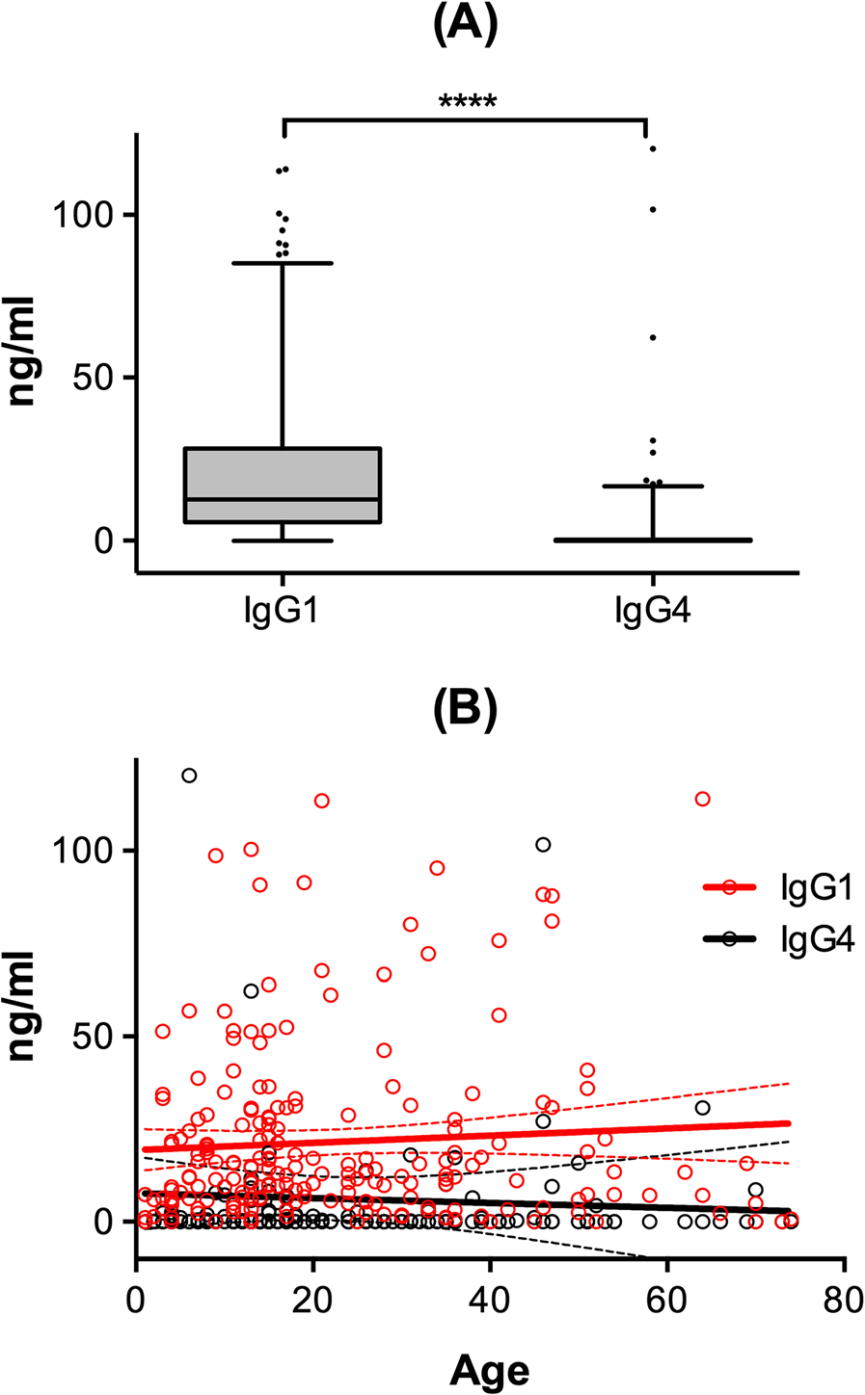
**Anti-cE5 IgG1 and IgG4 responses. (A)** Box plots of anti-cE5 IgG1 and IgG4 levels among the 207 individuals of the Aug 1994 survey. Boxes, whiskers, dots and P value as in Figure 1. IgG1 and IgG4 levels are expressed in ng/ml. **(B)** Scatter plot reporting the IgG1 (red) and IgG4 (black) antibody response to cE5 as function of age (n = 207). The best-fit lines (solid) and 95% CI (broken lines) are shown. Note that three data points are outside the axis limits.

Human IgG response to mosquito bites is known for being mainly characterized by saliva-specific antibodies of the IgG1 and IgG4 subclasses [28-30]. For this reason, and to obtain further insights into the human antibody response to the cE5 salivary protein, we also determined anti-cE5 IgG1 and IgG4 levels in the plasma of the same individuals previously analyzed (Aug 1994, n = 207). Median anti-cE5 IgG1 levels were significantly higher than IgG4 levels in exposed individuals (Figure 3A). This was independent of the specific age group considered (1–15, 16–30 or >30 years old, not shown; Wilcoxon test p < 0.0001) as clearly summarized by the scatter plot in Figure 3B. Therefore IgG1 appeared to be the dominant anti-cE5 IgG subclass, a finding substantially different from that previously reported for the response to the gSG6 protein, which is characterized by high IgG4 levels and by a switch from IgG1 to IgG4 taking place in children before ten years of age [26].

### Temporal variation of the anti-cE5 IgG response

**Figure 4:**
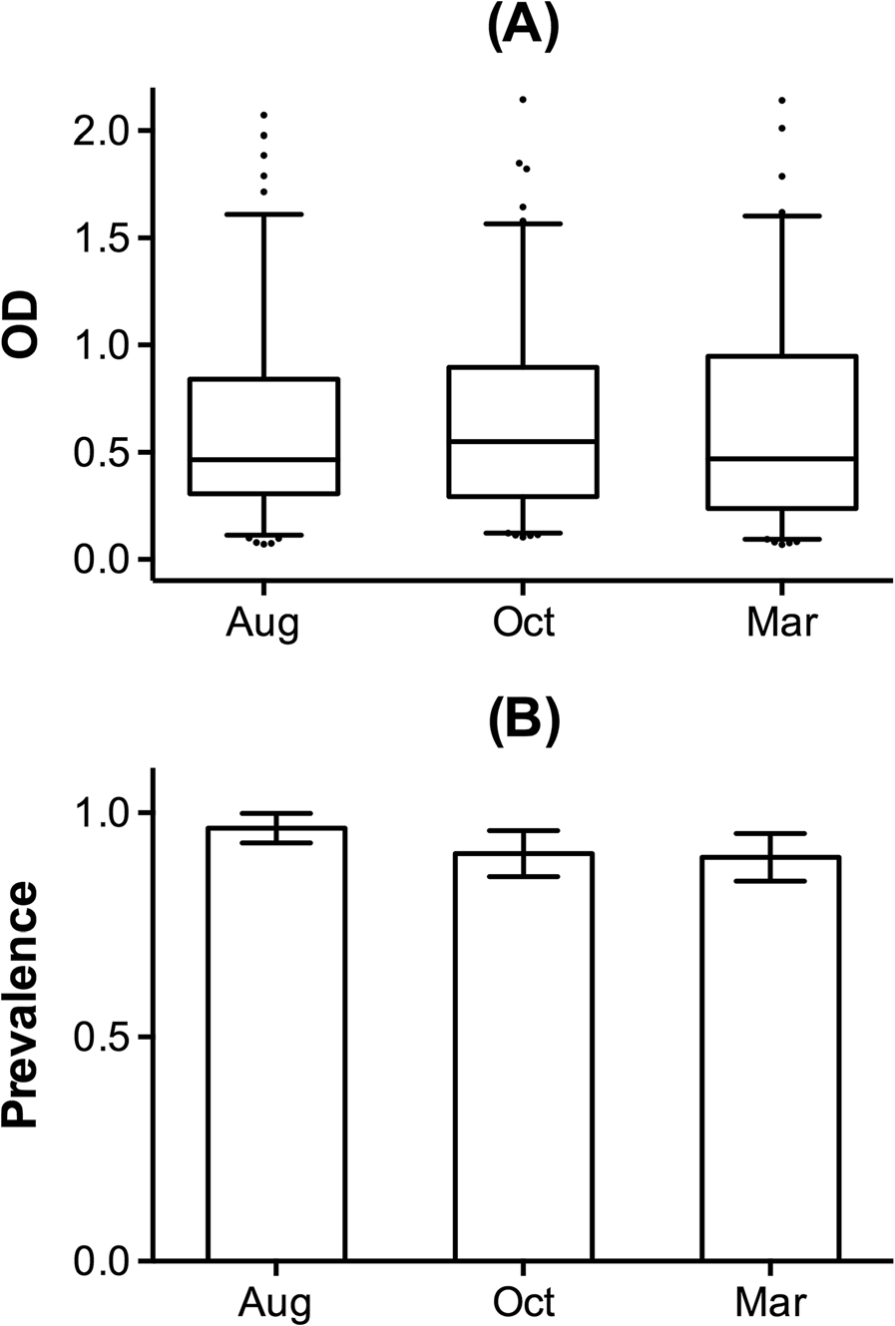
**Seasonal pattern of the anti-cE5 IgG response.** IgG response to the cE5 protein in the three surveys: Aug 1994 (n = 117, average age ±95% CI = 17.2 ± 3.0), Oct 1994 (n = 121, average age ±95% CI = 16.8 ± 3.1) and Mar 1995 (n = 121, average age ±95% CI = 16.0 ± 2.9). **(A)** Box plots of OD values among responders from the different surveys as indicated. Boxes, whiskers and dots as in Figure 1. IgG levels are expressed as OD_492_. **(B)** Seroprevalence of anti-cE5 IgG antibodies. Error bars as in Figure 1.

In order to better understand the kinetics of mounting and declining of the IgG response against the cE5 protein we measured anti-cE5 IgG antibodies in three sets of samples collected in the village of Barkoumbilen at the beginning (Aug 1994, n = 117) and at the end (Oct 1994, n = 121) of the high transmission/rainy season, as well as during the following low transmission/dry period (Mar 1995, n = 121). As also previously reported [15], in Aug and Oct 1994 vector density in the study area was high (number of anopheles/person/night was 6.3 ± 1.5 and 12.1 ± 5.4, respectively) while the number of anopheline mosquitoes showed a marked decrease during the dry season (0.4 ± 0.2 in Mar 1995). Comparison among cE5 responders indicated that IgG antibody levels did not change during the transmission season (Aug vs Oct), despite the continued exposure to anopheline bites. In addition, and more importantly, no decrease was observed during the dry season (Aug/Oct vs Mar) when a drop in vector density was recorded (Figure 4A). Identical results were obtained when the population was stratified in different age groups (not shown) and also seroprevalence was always very high (>90%) with no difference between the rainy and the dry seasons (Figure 4B). Similarly, no seasonal variation was observed when the comparison was restricted to the seventy-seven individuals for which plasma samples from the three different surveys were available. However, when the population was analyzed by age group, a significant decrease of the IgG level was found only in the group of youngest children*, i.e*. 1–5 years old (Additional file 2: Figure S2). Overall, these observations suggest that the anti-cE5 IgG antibody response is not short-lived and does not decay after a few months of very low or absent exposure as reported previously for both whole anopheles saliva [31] and the gSG6 antigen [15].

## Discussion

Mosquito salivary repertoires are known to include around one hundred secretory proteins and comparative analyses indicated that *Anopheles* mosquitoes carry in their saliva at least ten genus-specific proteins, *i.e*. not found in the saliva of *Aedes* or *Culex* species or in other blood feeding arthropods [1,10]. These proteins, if immunogenic to humans, may be ideal candidates for the development of innovative serological markers to evaluate human exposure to malaria vectors. We previously reported that one of these anopheline-specific salivary polypeptides, the *An. gambiae* gSG6, appeared to be a reliable marker of human exposure to Afrotropical malaria vectors as shown by studies in Burkina Faso, Tanzania and Uganda [13-16]. Moreover, peptides designed on the gSG6 protein were suitable to evaluate efficacy of insecticide-treated nets (ITN) for malaria vector control [32].

More recently we set up conditions for expression in recombinant form of the *An. gambiae* salivary anti-thrombin cE5 [22] and became aware of its immunogenicity. Considering the intrinsic variability of the individual immune response and the wide range of exposure to anopheline vectors in malaria endemic areas, it follows that the availability of additional markers would be very desirable. Therefore, we decided to compare the antibody response to cE5 and gSG6 in naturally exposed individuals from a malaria hyperendemic area of Burkina Faso. Among the 207 Rimaibé analyzed for their humoral response to both antigens we found ≈ 94% and ≈ 78% seropositivity with cE5 and gSG6, respectively (39 individuals responded only to cE5 and 6 only to gSG6). This finding clearly points to the high immunogenicity of cE5 which, along with the elevated vector density in the study area, explains the very high level of seropositivity.

To get some insights into the kinetics of the anti-cE5 antibody response we also measured specific IgG levels in samples collected at different time points: during the rainy season, *i.e*. in August and October when vector density is very high, and during the dry period (March) when there is a drop in vector density. No changes of the anti-cE5 IgG level or seroprevalence were observed. In the same epidemiological setting (identical village and surveys) we previously reported seasonal variation of the anti-gSG6 IgG response in individuals from the sympatric Mossi ethnic group: the IgG response to gSG6 increased from August to October with the continued exposure to anophelines and decreased in March, after a few months of very low or absent exposure [15]. The observations reported here clearly indicate that the cE5 *An. gambiae* salivary protein evokes in exposed individuals a relatively long-lasting antibody response, as opposed to the short-lived IgG response previously found for the gSG6 antigen. It should be pointed out that the sympatric ethnic groups Mossi and Rimaibé, who have been the subject of quite a few investigations related to genetic susceptibility to malaria, always showed very similar humoral response to all antigens tested so far [24,25]. For these reasons it seems unlikely that the difference in the response to these two antigens is connected to ethnicity, although this possibility cannot be completely ruled out.

It will be interesting to understand for how long the anti-cE5 IgG response persists in the absence of exposure to anophelines. This question could be addressed following subjects transiently exposed to bites of anopheline mosquitoes, for example travellers or workers spending a few months in tropical Africa. However, it should be taken into account that short-time exposures may not allow reaching a steady state, as suggested by the decrease of the anti-cE5 IgG response observed during the dry season in the eleven children under 5 years of age for which plasma samples were available from the three different surveys (Additional file 2: Figure S2). A possible alternative, in the absence of a suitable human challenge model, may be the use of a laboratory animal model (*i.e*. mice). In any event, the high sensitivity of the cE5 antigen suggests that it may be useful especially in situations of low vector density and perhaps to evaluate the impact of vector control measures such as the application of ITN or of long-lasting insecticide treated nets (LLIN).

The IgG antibody response to the cE5 and gSG6 salivary antigens also showed a striking difference in the way it evolves in individuals continuously exposed to anopheles bites throughout their life. The anti-cE5 IgG response increased with age, being less intense in children and reaching a maximum in adults after the age of thirty; this pattern is very similar to the one typically observed with different *P. falciparum* antigens in the same epidemiological setting [24,25]. On the contrary, as previously reported, anti-gSG6 IgG antibody levels tend to decrease according to age, most likely due to the induction of immune tolerance as also suggested by the high level of anti-gSG6 IgG4 antibodies and by the switch from IgG1 to IgG4 taking place in children [26]. A different pattern was found for the anti-cE5 IgG1 and IgG4 responses: IgG1 levels were always higher than IgG4 in all age groups, suggesting that the cE5 antigen does not induce immune tolerance and evokes an IgG1-dominated response, likely of the Th1-type.

In summary the results reported here clearly show that the two *An. gambiae* genus-specific salivary proteins gSG6 and cE5 trigger, in individuals naturally exposed to bites of anopheline mosquitoes, IgG antibody responses which are considerably different. The gSG6 protein evokes an IgG response of short-living nature, that is characterized by high anti-gSG6 IgG4 antibody levels and apparently inducing immune tolerance; on the contrary, the cE5 protein elicits a longer-living IgG antibody response, which does not seem to induce tolerance mechanisms and is characterized by higher concentration of anti-cE5 IgG1 antibodies. Predominant antigen-specific IgG1 and IgG4 antibody responses can be considered as indicators of Th1- and Th2-type polarization, respectively [33]. This consideration has some interesting implications since most arthropod-borne pathogens are introduced into vertebrate hosts in the context of vector saliva, whose immuno-modulatory properties may play relevant roles in pathogen transmission or establishment [5]. A common theme seems to be the ability of arthropod saliva to skew the host immune response by down-regulating Th1 cytokines (i.e. IFN-γ, IL-2) and up-regulating Th2 cytokines (i.e. IL-4, IL-6, IL-10) and creating an anti-inflammatory immunosuppressive environment that favors pathogen establishment as previously reported for ticks, sand flies and mosquitoes [34-39]. However, the history and timing of exposure to saliva may be critical as shown in a murine model of leishmaniasis. Indeed, sand fly saliva exacerbated *Leishmania* infection in naïve mice but had a protective effect in mice immunized by pre-exposure to bites of uninfected sand flies [34,40]. According to the most likely interpretation of these results, saliva immunization elicited a delayed-type hypersensitivity (DTH) Th1-type response at the bite site with early recruitment of immune cells and establishment of a pro-inflammatory environment unfavorable for *Leishmania* [34,40,41]. Moreover, also vaccination with individual sand fly salivary proteins inducing a DTH Th1-type response (with up-regulation of IFN-γ) was able to confer protection against *Leishmania* infection both in a mice and in a hamster model, indicating the possibility to exploit arthropod salivary proteins as a sort of adjuvants for vaccine development [42,43].

Only few studies on the effects of *Anopheles* mosquito saliva on *Plasmodium* transmission are reported in the literature, likely also due to the traditional assumption of malariologists that injected sporozoites rapidly abandon the inoculation site entering the blood stream to reach the liver. However, this view has recently changed: intravital fluorescence microscopy studies in murine models revealed that up to 50% of injected sporozoites stay in the skin, partly reaching draining lymph nodes and partly, before dying, going through further development up to merozoites in the dermis, epidermis or hair follicles [44-46]. These observations and recent studies on the early steps of *Plasmodium* infection of the mammalian host in murine models clearly pointed out that CD8+ T lymphocytes protective against malaria liver stages are primed in lymph nodes draining the inoculation site [47], highlighting the crucial role of the skin stage in malaria infection and immunity [48-50]. The possible effect of *Anopheles* saliva on immune response to *Plasmodium* parasites is still subject of debate. Some studies in murine malaria models showed that mice immunization by pre-exposure to bites of uninfected *An. stephensi* had a protective effect on the following infection with *P. yoelii* [51] or *P. chabaudi* [52]. In another investigation, however, *An. stephensi* saliva did not enhance infectivity of *P. yoelii* or *P. berghei* sporozoites and had no protective effect upon mice pre-immunization [53]. These contradictory observations may be, at least partly, explained by differences in the experimental systems. However, their interpretation is further complicated by the fact that blood feeding arthropod saliva is a complex cocktail whose components may have different and even contrasting effects (enhancing or suppressive) on host innate and adaptive immunity. Moreover, the specific effect may also be dose-dependent as the introduction into the skin of sub microgram amounts of antigens may induce immunosuppressive, antigen-specific T regulatory cells [48]. We believe that the use of individual salivary components as the *An. gambiae* cE5 and gSG6 proteins, rather than whole mosquito saliva, may be of great help in simplifying and standardizing the experimental systems and may contribute to shed some light on these intricate relationship between vector, parasite and vertebrate host.

## Conclusions

To our knowledge the *An. gambiae* gSG6 and cE5 are the first salivary proteins from an anopheline mosquito shown to trigger in naturally exposed individuals a qualitatively different immune response, most likely of the Th2- and Th1-type, respectively. We believe that the recombinant gSG6 and cE5 proteins described here may represent very useful reagents not only for malaria epidemiological studies to evaluate human exposure to malaria vectors but also to further investigate the involvement of *Anopheles* saliva/salivary proteins in the transmission and immunity to *Plasmodium* parasites.

## Additional files

Additional file 1: Figure S1. IgG response to cE5 and gSG6 in different age groups. Additional file 2: Figure S2. Anti-cE5 IgG response in individuals common to the three different surveys.

## Abbreviations

DTH: Delayed-type hypersensitivity
EIR: Entomological inoculation rate
HRP: Horse radish peroxidase
IFN-γ: Interferon-γ
IgG: Immunoglobulin G
IL-2: Interleukin 2
IL-4: Interleukin 4
IL-6: Interleukin 6
IL-10: Interleukin 10
ITN: Insecticide treated nets
LLIN: Long-lasting insecticide treated nets
OD: Optical density
OPD: O-phenylenediamine dihydrochloride
PBS: Phosphate buffered saline
PBST: Phosphate buffered saline plus 0.05% Tween-20
ROC: Receiver operating characteristic
