# Supplementary material for: Differential antibody response to the Anopheles gambiae gSG6 and cE5 salivary proteins in individuals naturally exposed to bites of malaria vectors
Source: Parasit Vectors. 2014 Nov 28;7:549. doi: 10.1186/s13071-014-0549-8 (PMC4253619; doi:10.1186/s13071-014-0549-8)
Supplement: Additional file 1: Figure S1. — IgG response to cE5 and gSG6 in different age groups. [file 13071_2014_549_MOESM1_ESM.pdf]

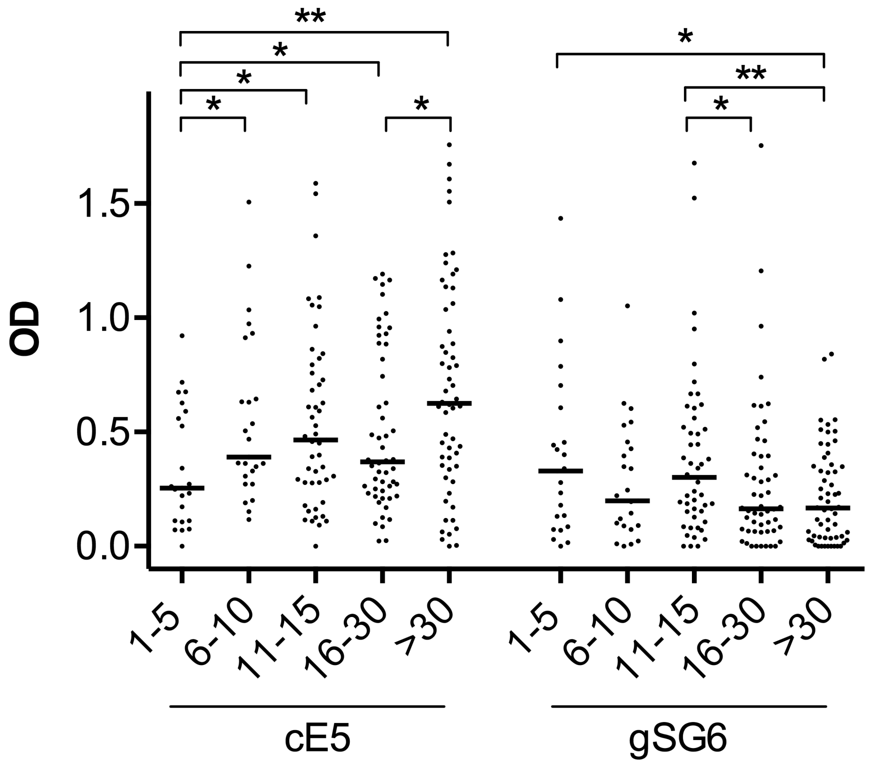

Figure S1. IgG response to cE5 and gSG6 in different age groups. Scatter plots reporting levels of IgG antibodies to the cE5 and gSG6 proteins in the 207 individuals stratified in five age groups as indicated (1-5 n=23; 6-10 n=25; 11-15 n=49; 16-30 n=52; >30 n=58). Bars represent median values. IgG levels are expressed as OD_{492}. P values were determined by the Kruskal-Wallis (cE5 p=0.0072; gSG6 p=0.0442) and the Mann-Whitney tests (*, p<0.05; **, p<0.01). Note that one data point is outside the axis limits.
