# Supplementary material for: Differential antibody response to the Anopheles gambiae gSG6 and cE5 salivary proteins in individuals naturally exposed to bites of malaria vectors
Source: Parasit Vectors. 2014 Nov 28;7:549. doi: 10.1186/s13071-014-0549-8 (PMC4253619; doi:10.1186/s13071-014-0549-8)
Supplement: Additional file 2: Figure S2. — Anti-cE5 IgG response in individuals common to the three different surveys. [file 13071_2014_549_MOESM2_ESM.pdf]

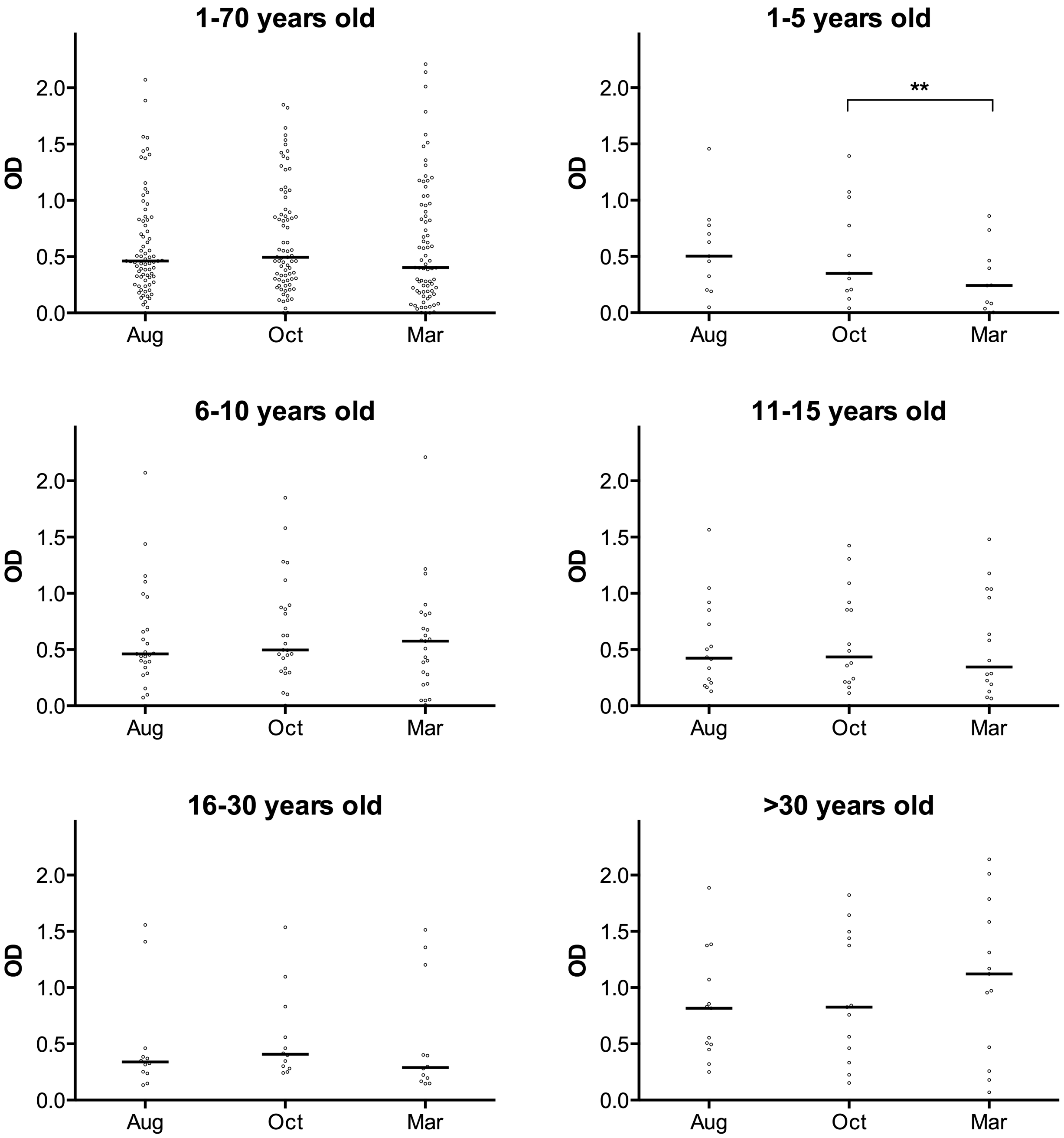

Figure S2. Anti-cE5 IgG response in individuals common to the three different surveys. Scatter plots reporting levels of IgG antibodies to the cE5 protein in the 77 individuals for which plasma samples were available in all three surveys. Plots refer to the whole cohort (top left) and to the five age groups as indicated. Bars represent median values. IgG levels are expressed as OD_{492}. Data were analyzed by the Friedman and Wilcoxon matched-pairs signed rank tests. Only statistically significant difference shown (**, $p<0.01$). Number of individuals was as follows: 1-5 years old, n=11; 6-10 years old, n=25; 11-15 years old, n=16; 16-30 years old, n=12; >30 years old, n=13.
